# Ionic Liquid-Assisted Acetylated Xylan Coatings Reinforced with CuO and ZnO Nanoparticles for Food Packaging Papers

**DOI:** 10.3390/polym18121527

**Published:** 2026-06-19

**Authors:** Petronela Nechita, Silviu-Marian Năstac

**Affiliations:** Research Centre for Mechanics of Machines and Technological Equipment—MECMET, Dunărea de Jos Unversity of Galați, 810017 Brăila, Romania; silviu.nastac@ugal.ro

**Keywords:** xylan, acetylation, ionic liquids, nanoparticles, packaging paper, barrier properties

## Abstract

This study investigates the potential of xylan acetylated using imidazolium-based ionic liquids, particularly 1-ethyl-3-methylimidazolium acetate ([Emim]Ac), as a functional matrix for ZnO and CuO nanoparticles (ZnO NPs and CuO NPs) in composite coatings for food packaging paper. A single coating layer (approximately 5 g/m^2^) was applied on both sides of the paper samples to improve barrier properties against water, oils, fats, and microbial contamination. The obtained results show that the combination of acetylated xylan with ZnO and CuO nanoparticles improved surface hydrophobicity, with contact angle values reaching 83° and 97°, respectively. The coatings exhibited antibacterial activity against *Bacillus* sp., as well as a reduction in fungal development of *Penicillium* spp., as evidenced by the observed inhibition of conidia sporulation. These findings indicate that ionic liquid-assisted acetylation of xylan using [Emim]Ac is an effective route for chemical modification of hemicelluloses. The developed xylan-based coatings demonstrate promising functional properties for potential application in sustainable food packaging materials, within the scope of the performed experiments.

## 1. Introduction

In recent years, the eco-friendly food packaging industry has experienced significant growth, driven by increasing environmental concerns and the continuously rising consumer demand for packaged food products [1]. In this context, natural polymers have attracted increasing attention for the development of films and coatings intended for food packaging applications due to their intrinsic properties, such as biodegradability and biocompatibility [2]. In general, films based on natural polymers exhibit barrier properties against gases, water, water vapor, oils, and fats, thereby contributing to the preservation of food quality and the extension of shelf life. In comparison with conventional synthetic polymers, packaging materials derived from renewable resources offer significant environmental advantages, including enhanced biodegradability, recyclability, and the potential for reuse [3]. Moreover, natural polymers provide an effective matrix for the incorporation of functional additives aimed at improving specific properties, such as antimicrobial activity or antioxidant capacity. Consequently, increasing attention has been focused on the use of natural polymers for the treatment and coating of paper intended for food packaging applications. Paper coating is considered one of the most effective approaches in the food packaging sector, as it significantly improves the functional properties of paper-based materials [4].

Polysaccharides constitute a broad class of natural polymers with considerable potential to replace synthetic polymers, owing to their non-toxic nature, biodegradability, good film-forming ability, and protective properties against oxygen and liquids. Among them, hemicelluloses represent the second most abundant group of polysaccharides after cellulose and are widely distributed in plant biomass, offering significant potential for valorization in the development of high value-added materials [5,6,7]. Xylan, the major component of hemicelluloses, accounts for the largest fraction and is widely present in hardwoods (approximately 30%) and softwoods (around 10%), as well as in various agricultural residues and as a by-product of the dissolving pulp manufacturing process [8,9].

A major limitation in the broad application of hemicelluloses is their pronounced hydrophilicity, which arises from the abundant free hydroxyl groups along the polymer backbone of their structural units [10]. For this reason, native xylan-type hemicelluloses display poor resistance to water, water vapor, oxygen, and microbial degradation [11,12,13]. Nevertheless, these hydroxyl groups also provide reactive sites for chemical modifications, allowing the introduction of hydrophobic functional groups. Such modifications can endow xylan-type hemicelluloses (xylan derivatives) with novel properties, thereby broadening their potential applications, particularly in food packaging, either as edible films or as coatings for packaging paper.

Numerous chemical reactions have been explored for the functionalization or modification of hemicelluloses, including oxidation, reduction, esterification (e.g., acetylation, propionylation, benzylation, or cross-linking), and etherification (e.g., cationization, carboxymethylation, or alkoxylation) [14,15,16]. These studies have shown that hemicelluloses can be efficiently esterified under homogeneous conditions, achieving a high degree of substitution. Generally, chemical modification of xylan through acetylation has been proposed as an effective strategy to reduce hydrophilicity and improve barrier properties. Acetylation of xylan introduces hydrophobic acetyl groups (–O–COCH_3_) in place of hydroxyl groups, thereby reducing the density of hydrogen-bonding sites available for interaction with water molecules. This chemical modification decreases the overall polarity of the polysaccharide and lowers its surface energy, resulting in enhanced hydrophobicity. As a consequence, acetylated xylan exhibits improved barrier performance when applied as a coating on paper substrates, leading to reduced water absorption and enhanced resistance to oils and moisture. These effects contribute to the formation of more efficient protective layers suitable for food packaging applications. However, such reactions often rely on organic solvents like dimethylformamide or pyridine, which are highly toxic and thus restrict the broad applicability of these methods. Consequently, there is a growing need to develop alternative approaches that provide comparable functionalization while minimizing environmental impact [17].

In this context, the use of ionic liquids, provides an efficient and more environmentally friendly reaction medium for the modification of hemicelluloses owing to their low vapor pressure, non-flammability, and high potential for recycling. Ionic liquids are substances composed entirely of ions that, unlike conventional salts, remain liquid at room temperature without the need for a molecular solvent. Research interest in ionic liquids emerged notably in the 1960s. Though there are extensive research and results on utilization of ionic liquids based on ammonium, pyridinium and imidazolium salts for cellulose modification, only a few are addressed to hemicelluloses and this topic becoming of high interest in the last period. By utilization of ionic liquids for hemicelluloses modifications with or without catalyst, high efficiency of reaction can be achieved. Furthermore, ILs offer a potentially clean method for carrying out chemical reactions or processes [18,19,20].

ZnO nanoparticles (ZnONPs) have emerged as one of the most widely used nanoparticles in industrial applications. Their structural characteristics confer important properties such as biocompatibility, high thermal stability, and notable antimicrobial activity, which contribute to their long-term durability. Owing to these advantageous features, ZnO nanoparticles have attracted considerable research interest, particularly in the development of food packaging materials based on ZnONPs and related nanocomposites [21].

CuO nanoparticles (CuONPs) have recently gained significant attention in food packaging due to their multifunctional properties and economic relevance. These benefits included enhanced water resistance, good mechanical performance, as well as antioxidant and antimicrobial characteristics, along with improved thermal and radiation protection [22]. Generally, CuO has ability to act as both electron donors and acceptors underpins their strong antimicrobial activity against a wide spectrum of foodborne contaminants. Moreover, the incorporation of CuONPs with other functional nanomaterials can further enhance packaging performance through synergistic interactions at the crystalline level [23].

The present study addresses the limitations of conventional xylan-based coatings by proposing a novel multifunctional coating system based on xylan hemicellulose esters. The innovation lies in the ionic liquid-assisted esterification of xylan using 1-ethyl-3-methylimidazolium acetate ([Emim]Ac), followed by the incorporation of CuO and ZnO nanoparticles to generate synergistic functional properties. This combined strategy enables the development of homogeneous composite coatings with enhanced antimicrobial activity and improved barrier performance. The coatings are applied as thin layers onto paper substrates, and their functional properties, including resistance to water, air, oils, and fats, as well as antimicrobial behavior are evaluated.

## 2. Materials and Methods

### 2.1. Materials

Xylan hemicellulose (purity ≥ 95% (HPLC)) derived from beechwood (Fagus sylvatica) purchased from Carl Roth GmbH (Karlsruhe, Germany) as powder form with a molecular weight of the repeating anhydroxylose unit of 132 g/mol, a melting point exceeding 300 °C, and a moisture loss on drying (3 h at 110 °C) of no more than 10.0%.

Ionic liquid based on imidazolium salts: 1-Ethyl-3-methylimidazolium acetate [Emim]Ac that is one of the most effective ionic liquids for xylan dissolution—with 95% purity, purchased from Sigma Aldrich (Taufkirchen, Germany).

ZnO nanoparticles (ZnO NPs), analytical grade, with an average particle size of approximately 100 nm (TEM), were purchased from Sigma-Aldrich (Taufkirchen, Germany) as a white aqueous dispersion with a solids content of 20 wt.%, density of 1.7 g/mL (25 °C), and pH 8.9 (20 °C).

CuO nanoparticles (CuO NPs), analytical grade, supplied as a black nanopowder with an average particle size below 40 nm (TEM), molecular weight of 79.55 g/mol, and density of approximately 6.32 g/cm^3^, were purchased from Sigma-Aldrich (Taufkirchen, Germany).

Reagents of analytical grade, including acetic anhydride (≥99% purity) and ethyl alcohol (≥99.5% purity, were purchased from Sigma-Aldrich (Taufkirchen, Germany) and used in the synthesis of xylan acetate.

The base paper used for coatings application was commercial grade, from unbleached pulp, and with a basis weight of 50 g/m^2^, with the main characteristics presented in Table 1.

### 2.2. Methods

#### 2.2.1. Synthesis of Xylan Acetate Using [Emim]Ac

**Figure 1:**
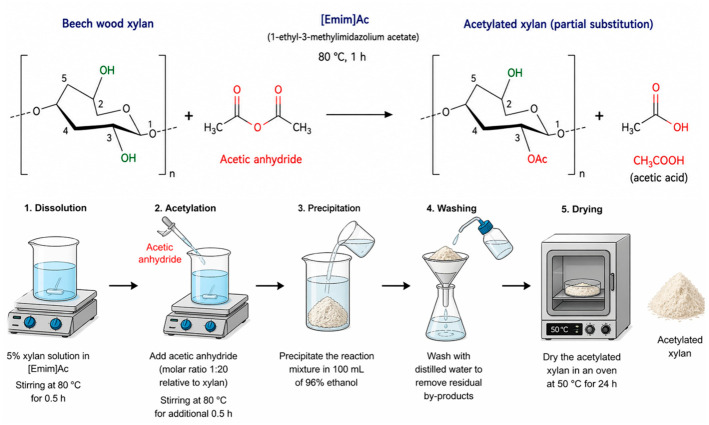
Chemical reaction and schematic diagram of xylan acetylation.

For the chemical modification of beech wood xylan, the following procedure was used: a solution of xylan and 1-Ethyl-3-methylimidazolium acetate at a concentration of 5% is stirred for 40 min at a temperature of 80 °C. Then, acetic anhydride is added in a molar ratio of 1:20 relative to xylan. Stirring was continued at the same temperature for an additional 0.5 h. The resulting mixture was precipitated in 100 mL of 96% ethanol and then washed with distilled water to remove residual reaction by-products. The obtained acetylated xylan with degree of substitution (DS) of 0.87, was dried in an oven at 50 °C for 24 h (Figure 1).

The degree of substitution (DS) of acetylated xylan was determined by titration. Approximately 0.1 g of acetylated xylan was mixed with 8.0 mL of 0.25 M NaOH and 5.0 mL of ethanol, and the mixture was stirred for 24 h. Subsequently, 15.0 mL of 0.25 M HCl was added, and the mixture was allowed to stand for 30 min. The excess acid was then titrated with 0.25 M NaOH using phenolphthalein as an indicator.

#### 2.2.2. Structural Analysis (FT-IR, ^1^H-RMN, SEM-EDX)

Native and acetylated xylan were structurally characterized by FT-IR spectroscopy using a Nicolet iS50 instrument (Thermo Fisher Scientific, Waltham, MA, USA) fitted with an attenuated total reflectance (ATR) module. The spectra were obtained over the range of 4000–400 cm^−1^ by averaging 32 scans at a spectral resolution of 4 cm^−1^.

Further structural confirmation was performed by ^1^H-NMR spectroscopy using a Bruker^®^ Avance DRX 400 spectrometer (Bruker, Billerica, MA, USA). This method relies on the excitation of hydrogen nuclei (^1^H) by radiofrequency pulses and the subsequent Fourier transformation of the generated response. Proton spectra were recorded at an operating frequency of 400 MHz. Dimethyl sulfoxide (DMSO) served as the solvent, while all measurements were carried out at 60 °C.

The morphology of paper surfaces coated with the composite formulations was investigated by scanning electron microscopy (SEM) FEI QUANTA 200^®^ (Thermo Fisher Scientific, USA). The analyses were performed under vacuum conditions in order to preserve beam stability and avoid disturbances caused by air particles. The interaction between the electron beam and the sample surface generated signals that enabled the evaluation of both surface structure and elemental composition. To ensure complete characterization, specimens were prepared from both sides of each paper sample. Prior to analysis, the samples were fixed on aluminum holders with conductive carbon tape and coated with a thin gold film using a plasma sputtering device (SPI Sputter Coater Module, SPI Supplies, West Chester, PA, USA). Micrographs were captured with an FEI QUANTA 200^®^ scanning electron microscope (Thermo Fisher Scientific, USA) coupled with an EDAX 32 elemental analysis system, using a magnification of 1000× and a scale bar of 100 µm. The working distance was maintained at 10 mm, the accelerating voltage was set to 15 kV, and secondary electrons were used for signal detection to maximize elemental excitation [24].

#### 2.2.3. Thermal Stability Characterization

The thermal behavior of native xylan and chemically modified acetylated xylan was investigated by thermogravimetric analysis (TGA) using a Discovery TGA 5500 Thermogravimetric Analyzer (TA Instruments, New Castle, DE, USA) under a controlled atmosphere. This technique was applied to determine the thermal degradation profile, including the decomposition temperature, the different stages of thermal degradation, and the corresponding mass loss at selected temperatures. For each analysis, approximately 10 mg of sample was heated from 25 °C to 700 °C at a heating rate of 10 °C/min. The obtained data were further analyzed using TA Instruments Universal Analysis 2000 software.

#### 2.2.4. Preparation of Composite Coatings

The coating formulation was established based on previous optimization studies conducted by the authors, which identified 2.5 wt.% xylan as the optimum concentration for obtaining homogeneous coatings with suitable viscosity and film-forming properties. Nanoparticle contents of 10–20 wt.% (based on xylan dry weight) were selected to maximize barrier and antimicrobial performance while avoiding particle agglomeration [24,25].

The composition of each coating layer and the coding of the paper samples coated with the composite mixtures are presented in Table 2.

#### 2.2.5. Preparation of Coated Papers

**Figure 2:**
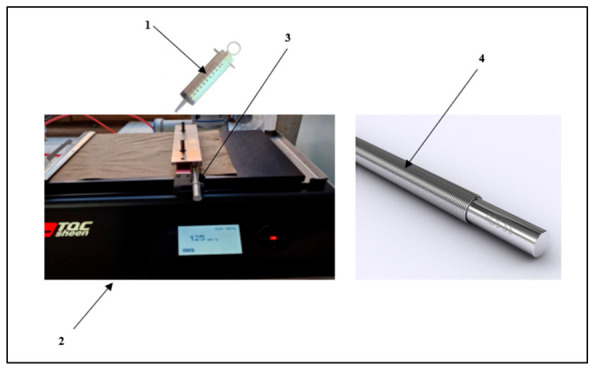
Process diagram for the application and leveling of the composite mixture on the surface of the paper. 1. Manual dosing device for the coating mixture; 2. Automatic application device for the composite mixture. 3. Mayer rod for leveling and equalizing the coating layer. 4. Detail of the Mayer rod.

Colloidal dispersions of xylan, acetylated xylan, and composite coatings with ZnONPs and CuONPs were applied to the surface of the base paper in a single layer on each side of the paper, at predetermined doses to ensure a coating layer of 4.5–5.0 g/m^2^ for each side of paper sample. The coating process was carried out using a TQC SHEEN automatic film applicator (TQC B.V., Capelle aan den Ijssel, The Netherlands). In this technique, the aqueous dispersion is placed in front of a wire-wound bar, which moves automatically across the paper surface to provide a homogeneous coating layer. The bar coater is made of stainless steel, with a total width of 290 mm and a spiral-wound metal coating width of 230 mm, ensuring the application of a coating layer with a thickness of 40 μm (Figure 2). 

In total, 20 paper samples with dimensions of 20 × 25 cm were coated and analyzed for their functional performance. All the tests and analyses in the experimental program were performed in triplicate, and the reported results are expressed as mean values ± standard deviation. The paper treated with native xylan only, was used as reference material throughout the study.

#### 2.2.6. Functional Properties of Coated Papers

- Barrier properties

Air Permeability—commonly referred to as “Gurley Porosity,” represents the time required for a fixed volume of compressed air (100 mL) to pass through a defined area of the sample (6.42 cm^2^). The parameter is expressed as seconds per 100 mL (s/100 mL), with higher values indicating lower air permeability. This property was determined directly using a Genuine Gurley™ Model 4340 Automatic Densometer (Gurley Precision Instruments, Troy, NY, USA), following the ISO 5636-5:2013 standard [26].

Water Contact Angle was determined using the Sessile Drop method in accordance with the TAPPI T 458 cm-04 standard (2004) [27], employing the Ossila Contact Angle Goniometer (Ossila BV, Leiden, The Netherlands, 2022, Model L2004A1.1, measuring range 5–180°). During the analysis, the paper specimen was fixed on the testing platform of the goniometer using clamps, and 3 µL droplets of distilled water were carefully placed on the surface with a micro syringe from a height of 2.5 mm. The contact angle of each droplet was measured after 5 s of contact between water and paper, and the obtained values were processed using the dedicated analysis software Ossila, version 4.0. [27].

The resistance of paper samples to oils and fats was evaluated using the KIT Test, according to the TAPPI T559-cm12 standard [28]. This method involves assessing the interaction between the paper surface and a set of standard test solutions assigned KIT numbers from 1 to 12 [28]. These solutions consist of different proportions of castor oil, toluene, and n-heptane. For the analysis, paper specimens were cut to dimensions of 51 mm × 152 mm (width × length), and both sides (F/S) were properly identified. Each sample was placed on a clean, flat, and well-illuminated surface with the side to be tested facing upward. The procedure began with the application of a drop from a solution with an intermediate KIT number, carefully deposited from a height of 13 mm using a pipette, while the timing was started immediately. After 15 s, the tested area was visually examined. If the drop penetrated the paper surface and produced a visible stain, the sample was considered to have failed at that KIT value, and the test was repeated using a lower KIT number solution. If no stain was observed and the drop remained stable on the surface, the sample was considered to have passed, and the procedure continued with the next higher KIT number. The test was repeated progressively until the first visible stain appeared, which indicated the maximum KIT value supported by the paper sample.

- Assessment of antibacterial and antifungal activity

The antimicrobial performance of the coated paper samples was evaluated against Gram-positive bacteria (*Bacillus* sp.) and fungi (*Penicillium* sp.) obtained from the MIUG Collection of the BioAliment Research Platform, “Dunărea de Jos” University of Galați, Romania.

The antibacterial activity was determined using a modified procedure based on the SR EN ISO 846/2000 standard [29]. Prior to testing, the paper samples coated with xylan and xylan derivatives and chitosan were sterilized by ultraviolet (UV) exposure for 15 min. Afterwards, the specimens were placed on the surface of Plate Count Agar (PCA) culture medium (Merck Millipore, Darmstadt, Germany). Inoculation was performed by applying 1 µL of bacterial suspension (10^6^ CFU/mL), previously cultured for 18 h, onto the coated paper surfaces. The samples were incubated at 37 °C in a thermostat and evaluated after 24 and 48 h. The degree of bacterial growth was assessed by assigning a score defined as follows: very good growth (+ + +), good growth (+ + −), poor growth (+ − −), and no growth (− − −), which inversely correlates with the inhibition potential of the analyzed paper samples, or by calculating the inhibition percentage of the coated surface, based on the extent of bacterial growth observed on and around the samples. According to the mentioned standard, the greater degree of microbial growth (i.e., the more numerous the colonies), the lower the antimicrobial activity score [29].

The antifungal activity was investigated using fungal suspensions of approximately 10^7^ CFU/mL cultivated on Rose Bengal culture medium (Merck, Germany). The nutrient medium surface was first flooded with the conidia suspension, followed by placing the paper specimens directly on top of the inoculated medium. The incubation was carried out at 25 °C and 85% relative humidity for 21 days. Fungal growth was monitored after 3, 7, 14, and 21 days and was expressed as the percentage of the paper surface covered by fungal colonies.

## 3. Results and Discussion

### 3.1. FT-IR Analysis

**Figure 3:**
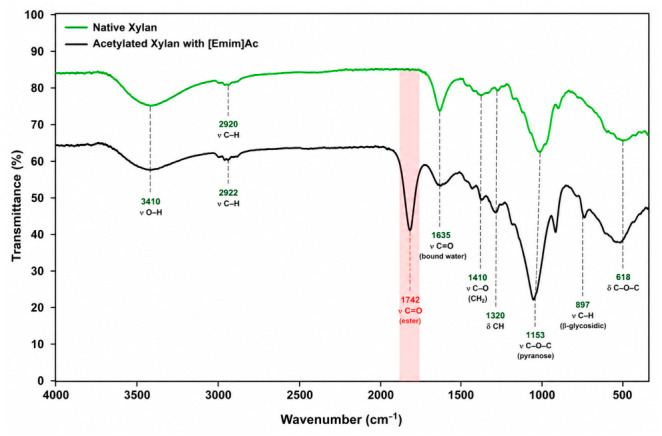
FT-IR spectra of native and acetylated xylan.

The analysis of the FT-IR spectra for the two types of xylan presented in Figure 3 revealed the following aspects: the absorption bands at 3360–3330 cm^−1^, 2880, 1602, 1456, 1250, 1161, 1032, 980, and 895 cm^−1^ are characteristic of xylan-type hemicelluloses. The broad absorption band at 3360–3330 cm^−1^ is attributed to the stretching vibrations of the –OH groups from the structural unit of xylan. The decrease in intensity at 3360–3330 cm^−1^ is associated with the reduction in hydroxyl groups in xylan as a result of chemical modification during the esterification process. The absorption band at 2913 cm^−1^ is assigned to the stretching vibration of aliphatic C–H groups. The stretching vibrations of C–O–C and C–O groups, together with a certain contribution of the OH bending mode, may contribute to the band at 1160 cm^−1^. The prominent band at 1034–1043 cm^−1^ corresponds to the stretching vibrations of C–O–C groups in the pyranose ring. A sharp band at 896 cm^−1^ originates from the β-glycosidic linkages between xylose units. In contrast, the intense signals at 1742 cm^−1^ and 1635 cm^−1^ in the spectrum are attributed to the stretching vibrations of C=O groups (ester groups) [30,31]. These results confirm that acetylation was successfully achieved, while the increased intensity of the band at 1742 cm^−1^ for the samples modified with [Emim]Ac indicates the degree of acetylation (Figure 3).

### 3.2. ^1^H-NMR Analysis

**Figure 4:**
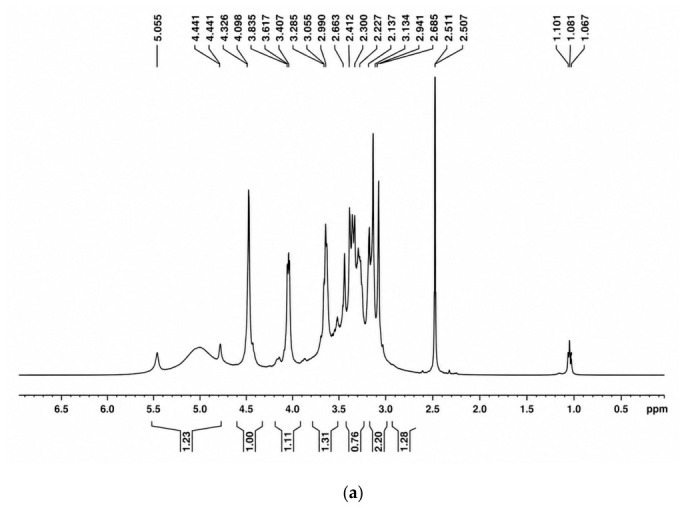

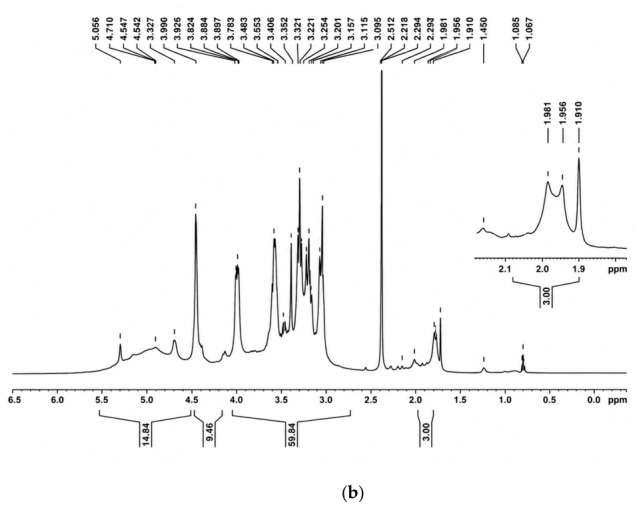
^1^H-NMR spectra of: (**a**) beechwood native xylan; (**b**) beechwood xylan acetylated with [Emim]Ac ionic liquids.

The ^1^H-NMR spectrum shows intense signals corresponding to the protons of xylose units from the unsubstituted main chain, as well as less intense signals attributed to hydroxyl groups. The signals observed in the ^1^H-NMR spectra (Figure 4a,b) in the range of 3.3–5.0 ppm are assigned to the protons of the xylan ring. For acetylated xylan (Figure 4b), the strong signal at 2.0 ppm is characteristic of the protons from acetyl groups (–CH_3_–CO–), confirming the acetylation of xylan hemicellulose [25].

In the spectrum of acetylated xylan (Figure 4b), the characteristic signals of the basic xylan structure are still present in the 3.0–5.0 ppm region, indicating that the main polysaccharide backbone was not degraded during the reaction. However, new intense signals appear in the 1.9–2.1 ppm region, which are attributed to the methyl protons (–CH_3_) of the acetyl groups introduced through the esterification reaction. The presence of these signals represents the main evidence of xylan acetylation. In addition, a slight decrease in the intensity of the signals associated with hydroxyl protons and changes in the integral ratio of the signals can be observed, supporting the partial substitution of –OH groups with acetyl groups. The increased intensity of the signal around 2.0 ppm and its higher ratio compared to the proton signals of the xylan backbone indicate a high degree of acetylation for the sample modified with [Emim]Ac.

These results confirm the successful acetylation reaction and demonstrate the efficiency of the ionic liquid [Emim]Ac both as a solvent and as a favorable reaction medium for the chemical modification of xylan.

### 3.3. Thermal Stability Analysis

**Figure 5:**
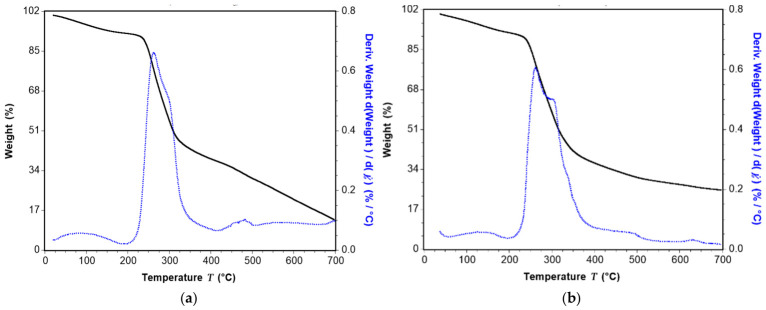
Thermal stability of (**a**) native xylan and (**b**) acetylated xylan.

From the TGA results presented in Figure 5, a small initial mass loss is observed for both samples in the range of 25–100 °C, which is attributed to the removal of physically adsorbed water. Native xylan (Figure 5a) shows a main thermal degradation step starting at approximately 305 °C, corresponding to the decomposition of the polysaccharide backbone. For acetylated xylan (Figure 5b), an additional weight loss stage is observed in the range of approximately 200–280 °C, which is attributed to the deacetylation process and release of acetic acid. The main degradation of the polysaccharide backbone also occurs at around 300–310 °C, indicating a comparable thermal stability of the xylan structure after modification. In some cases, a higher residual mass at elevated temperatures can be observed for the modified sample, which may be associated with the formation of a more thermally stable char structure. The apparent differences in thermal behavior can be related to the reduction in free hydroxyl groups after acetylation, which decreases hydrogen bonding interactions and slightly modifies the degradation pathway [25].

### 3.4. Functional Properties

Overall, the application of composite coatings based on xylan derivatives and ZnO/CuO nanoparticles led to an enhancement of the functional properties of the paper samples compared to the untreated base paper (Table 1). The results obtained for the coated papers revealed improved barrier and surface properties, highlighting the beneficial effect of the proposed coating formulations.

**Figure 6:**
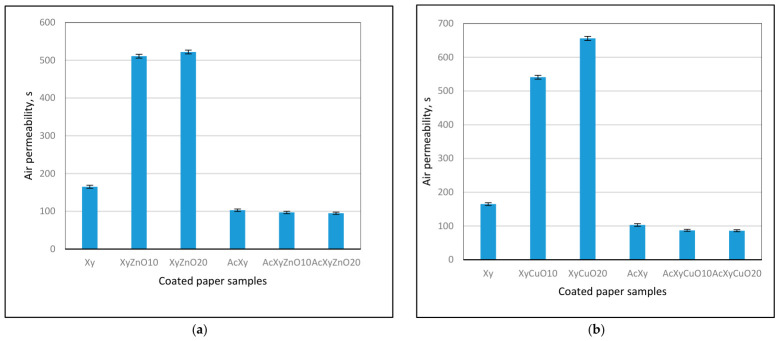
Air permeability of paper coated with xylan hemicellulose and metallic oxides nanoparticles: (**a**) xylan and acetylated xylan with ZnONPs; (**b**) xylan and acetylated xylan with CuONPs; Data are presented as mean ± sdv (n = 3).

Barrier properties are among the main functional characteristics of food packaging materials. A high level of these properties helps maintain quality and extend the shelf life of packaged food. In Figure 6a,b is presented the air permeability of coated papers with composite coatings based on native/acetylated xylan and ZnONPs/CuONPs. It is observed that the samples coated with acetylated xylan (AcXy) indicate a higher air permeability (the lower air penetration times recorded) compared to those treated with native xylan (Xy), even at constant coating thickness and nanoparticle loading. This behavior can be attributed to differences in intermolecular interactions, film-forming ability, and nanoparticle integration within the coating matrix. By acetylation the number of available hydroxyl groups is reduced, limiting hydrogen bonding interactions both within the xylan matrix and at the fiber–coating interface. As a result, acetylated xylan exhibits weaker adhesion to the cellulose substrate and a reduced ability to form continuous and compact films. Consequently, the coating layer is more prone to structural discontinuities and incomplete pore sealing, leading to a more open structure and increased air permeability. The presence of ZnO and CuO nanoparticles further accentuates this effect. In the case of acetylated xylan, the weaker polymer–fiber and polymer–polymer interactions hinder the uniform embedding of nanoparticles within the matrix. This can lead to the formation of microvoids or interfacial gaps around the particles, which act as additional pathways for air diffusion. In contrast, native xylan, with its higher density of hydroxyl groups, promotes stronger intermolecular cohesion and better anchoring of nanoparticles, resulting in a more homogeneous and compact structure with reduced permeability. The decreased air permeability enhances the barrier performance, which is beneficial for food packaging by reducing oxygen transmission and improving shelf-life stability.

The variations in air permeability of tested paper samples are further confirmed by SEM micrographs of coated paper samples with acetylated xylan and nanoparticles of ZnO and CuO.

**Figure 7:**
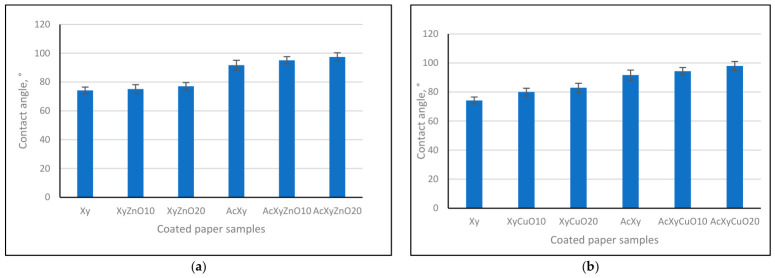
Contact angle values of paper coated with xylan hemicellulose and metallic oxides nanoparticles: (**a**) xylan and acetylated xylan with ZnONPs; (**b**) xylan and acetylated xylan with CuONPs; Data are presented as mean ± sdv (n = 3).

The results obtained for contact angle indicate a significant improvement in surface hydrophobicity for paper samples coated with acetylated xylan and ZnO and CuO nanoparticles (approx. 97°), compared to those coated with native xylan dispersions and metal oxide nanoparticles (about 77–83°) (Figure 7a,b). The increase in contact angle values for acetylated xylan-based coatings is primarily due to the acetylation process when the substitution of hydroxyl groups with less polar acetyl groups occurs [32]. In contrast, native xylan retains its hydrophilic character due to the abundance of hydroxyl functional groups. The incorporation of metal oxide nanoparticles (ZnO and CuO) further influences the surface wettability, likely through changes in surface roughness and polymer–particle interactions. However, in acetylated systems, which have a denser coating layer compared with native xylan (SEM images presented in Figure 9) coated samples, the chemical modification plays a dominant role, resulting in similarly high contact angle values regardless of nanoparticle type.

**Figure 8:**
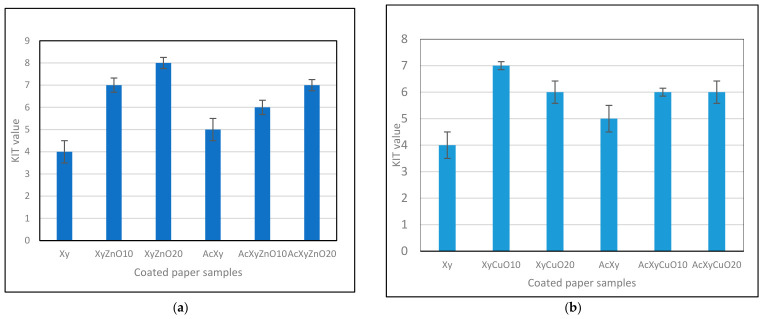
Oils and fats resistance of paper coated with xylan hemicellulose and metallic oxides nanoparticles: (**a**) xylan and acetylated xylan with ZnONPs; (**b**) xylan and acetylated xylan with CuONPs.

The resistance to oils and fats is other property that is very important of food packaging. In Figure 8a,b is presented the variation in this parameter with the composition of coating formulas. It is observed that the incorporation of ZnO nanoparticles in native xylan matrix, significantly improved the KIT performance, with values increasing to 7 for XyZnO10 coated samples and reaching the maximum value of 8 for XyZnO20 coated samples, comparing with native xylan reference coated paper where the KIT value is 4. This behavior suggests that metal oxide nanoparticles contributed to the formation of a denser and more uniform coating layer, reducing surface porosity and limiting the diffusion of oily substances through the paper structure. In the case of samples coated with acetylated xylan (AcXy), the initial KIT value increased to 5, confirming that the acetylation improved the hydrophobic character of the coating due to the partial substitution of hydroxyl groups with acetyl groups [33]. When ZnO nanoparticles were incorporated into the acetylated xylan matrix, the KIT values further increased to 6 for AcXyZnO10 samples, and 7 for AcXyZnO20 samples, indicating a synergistic effect between chemical modification and inorganic nanoparticle reinforcement.

For the CuO-based formulations, a similar trend was observed, although the improvement was slightly less pronounced compared to ZnO. Native xylan presented a KIT value of 4, while XyCuO10 samples reached 7 and XyCuO20 samples showed a slightly lower value of 6. This suggests that, at higher CuO concentrations, partial nanoparticle agglomeration may occur, reducing coating uniformity and slightly decreasing barrier efficiency [22]. For acetylated xylan samples, the KIT values increased from 5 (AcXy) to 6 for both samples, AcXyCuO10 and AcXyCuO20, indicating a positive but more moderate effect of CuO addition.

Overall, the results indicate that both acetylation and nanoparticle incorporation improved the grease resistance of paper, the samples coated with acetylated xylan and ZnONPs showing a stronger positive effect than those coated with acetylated xylan and CuONPs. In addition, considering that KIT values for commercial grease-proof papers obtained with fluorochemicals or other functional coatings are typically in the range of 4–10 [34,35], the values obtained in this study are considered promising for potential food packaging applications. These coatings are based on renewable, biodegradable, and highly recyclable materials, which further supports their sustainability advantages.

### 3.5. Structural Morphology of Coated Papers Surface

**Figure 9:**
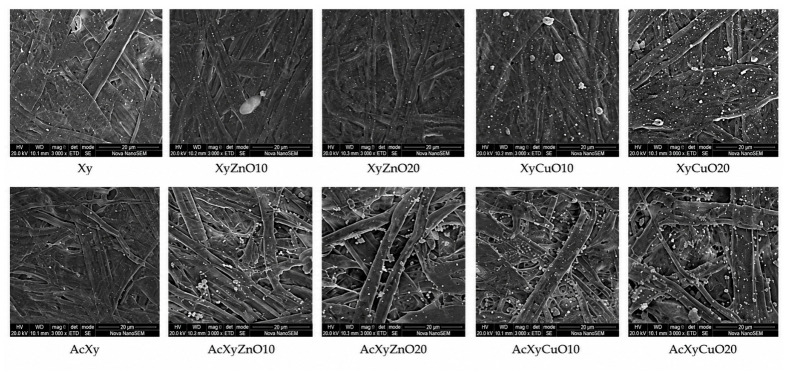
SEM micrographs of paper coated with xylan hemicellulose and ZnO/CuO nanoparticles.

SEM micrographs presented in Figure 9 indicate that the composite coatings do not form a fully continuous film, preserving the fibrous structure of the paper substrate. While native xylan-based coatings exhibit relatively better surface coverage and cohesion, acetylated xylan systems show a more heterogeneous morphology, characterized by microvoids, discontinuities, and less uniform nanoparticle distribution. These structural features promote the formation of preferential pathways for air flow, which is in agreement with the higher air permeability observed for acetylated xylan-based samples in previous Figure 6.

**Figure 10:**
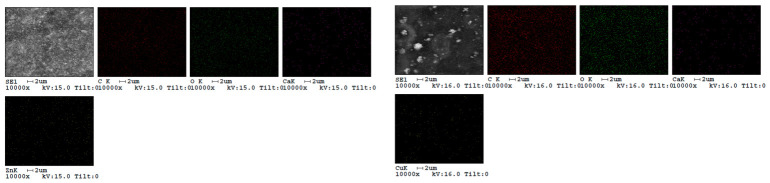

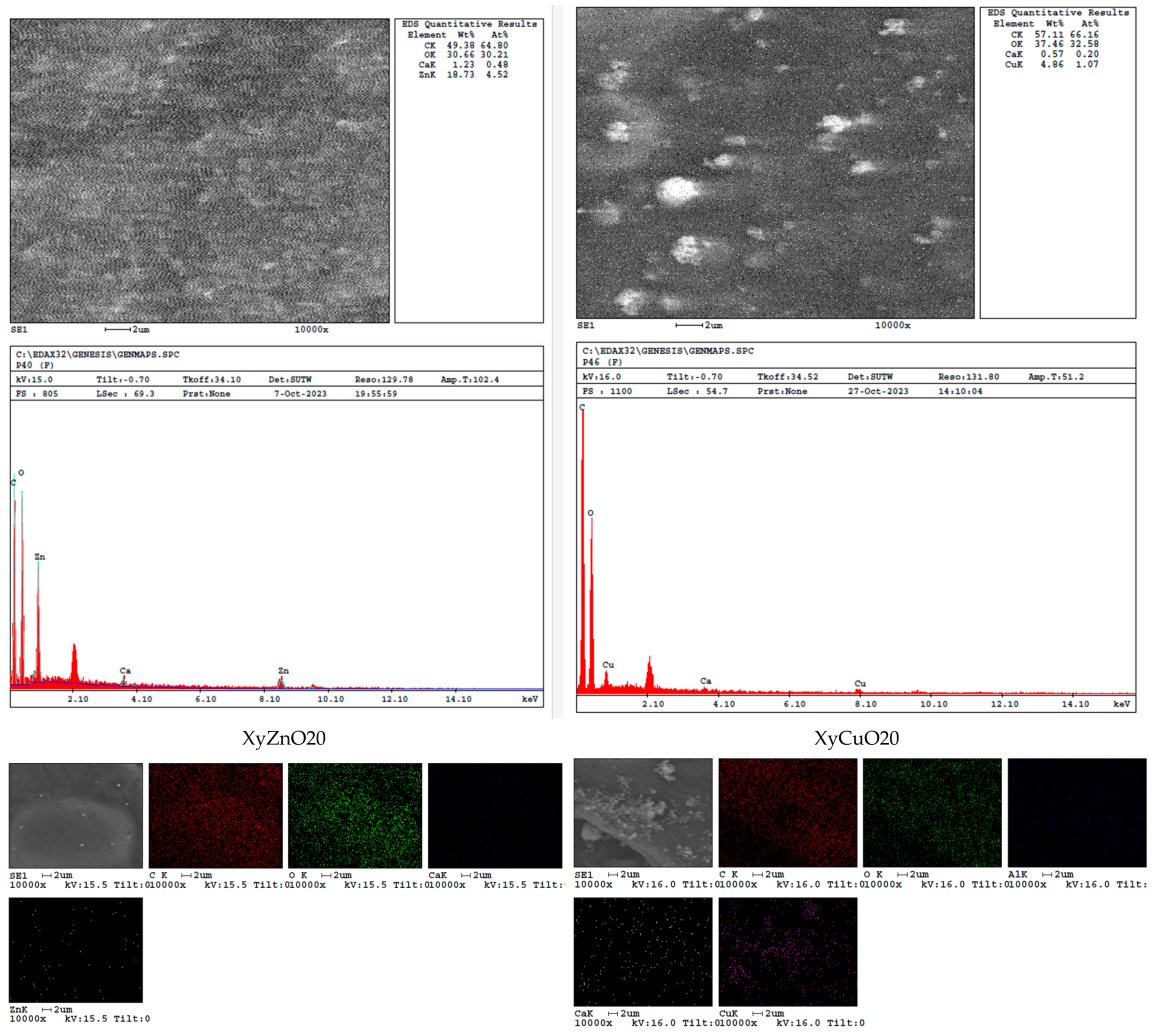

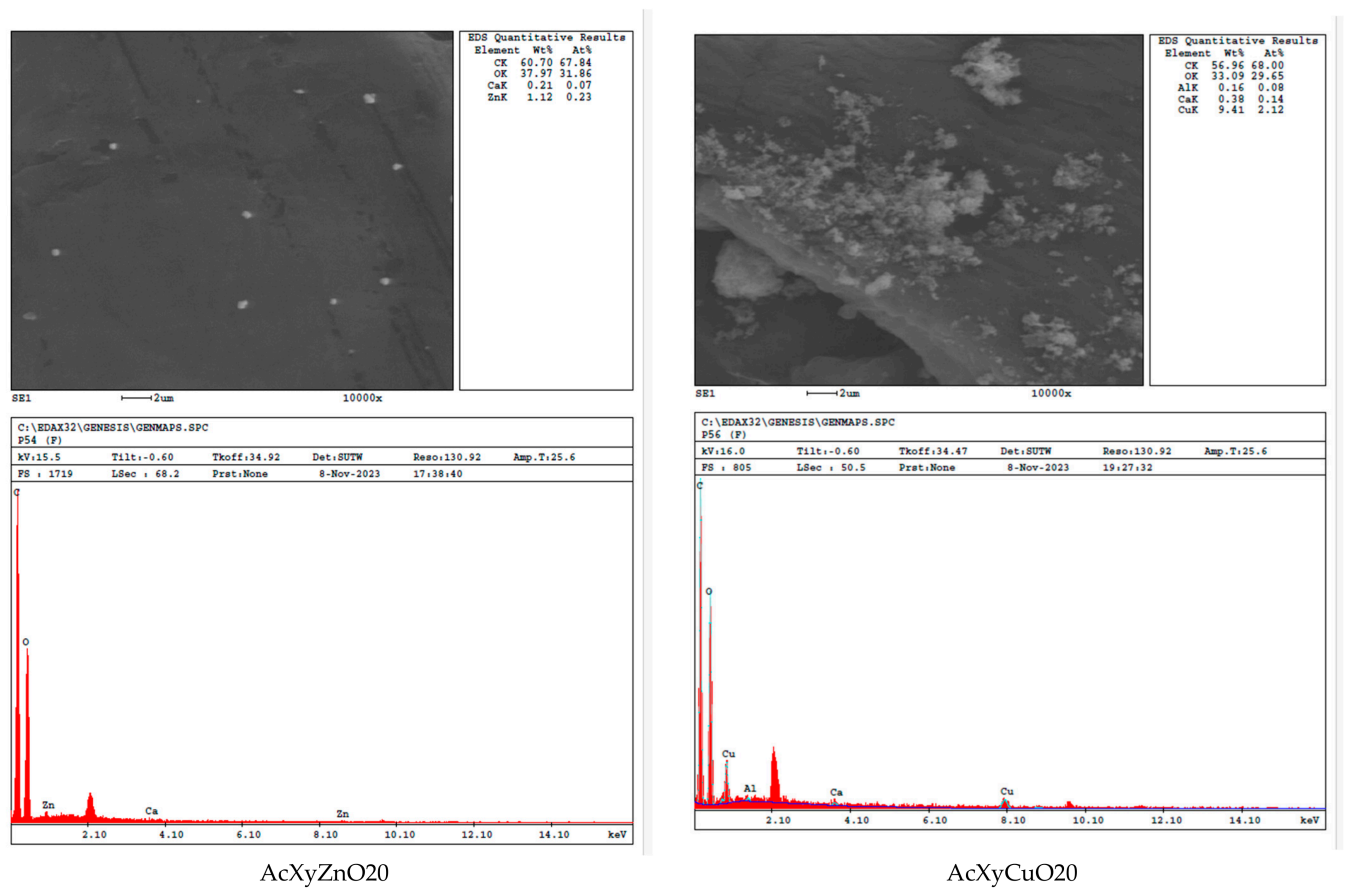
EDX analysis of papers coated with composite based on xylan hemicellulose and ZnO/CuO nanoparticles.

This structure is confirmed by EDX elemental mapping (Figure 10) where a tendency for aggregation of ZnO/CuO nanoparticles can be observed in the composite mixtures. This tendency is more pronounced in the case of systems based on acetylated xylan and CuO nanoparticles. This behavior may be attributed to their smaller particle size (<40 nm), compared to ZnO nanoparticles (<100 nm), as well as to their less uniform incorporation within the acetylated xylan dispersions.

### 3.6. Antibacterial and Antifungal Activity

The antibacterial activity of the coated paper samples (Table 3) strongly depends on both the chemical modification of xylan and the presence of metal oxide nanoparticles. The samples coated with acetylated xylan (AcXy) exhibited moderate antibacterial activity (approx. 40% inhibition), likely due to increased surface hydrophobicity and reduced bacterial adhesion. In contrast, the samples coated with native xylan (Xy) showed no inhibitory effect. The incorporation of ZnO nanoparticles significantly enhanced antibacterial performance, leading to complete inhibition (100%) in the case of samples coated with native xylan-based coatings containing 20% ZnO (XyZnO20) [36]. This effect can be attributed to improved nanoparticle dispersion and accessibility within the hydrophilic matrix. Conversely, the samples coated with acetylated xylan-based composites containing ZnO (AcXyZnO10 and AcXyZnO20 and) showed lower inhibition (66% and 50%), likely due to reduced diffusion and limited interaction between nanoparticles and bacterial cells. CuO-containing samples exhibited strong initial antibacterial activity (66% after 24 h), followed by a decrease over time, possibly due to reduced ion release and diffusion limitations [37]. Overall, the results highlight a trade-off between matrix hydrophobicity and nanoparticle bioavailability in determining antibacterial efficiency.

The results on antifungal activity are presented in Table 4. Although no inhibition of mycelial growth (0% inhibition rate) was observed for all tested samples over the entire incubation period, a pronounced inhibitory effect on sporulation was detected. This behavior indicates that the coatings exert a sublethal antifungal effect, primarily affecting fungal differentiation rather than vegetative growth. In the case of native xylan-based coatings containing ZnO nanoparticles, sporulation inhibition observed after 3 days may be attributed to transient nanoparticle-induced stress. In contrast, acetylated xylan-based coatings exhibited consistent inhibition of sporulation, likely due to increased surface hydrophobicity, which alters the microenvironment at the material–fungus interface. The combination of acetylated xylan with ZnO and CuO nanoparticles resulted in a more persistent inhibitory effect on sporulation, suggesting a synergistic interaction between surface hydrophobicity and nanoparticle-induced oxidative stress.

## 4. Conclusions

The performance of acetylated xylan with ionic liquids based on imidazolium salts, was evaluated after its incorporation into composite formulations containing ZnO and CuO nanoparticles, which were applied as thin coating layer on the paper surface (approximately 5 g/m^2^). The developed coatings impart improved barrier properties against water, oils, fats, and microbial attack, to packaging paper.

For paper samples coated with acetylated xylan combined with ZnO and CuO nanoparticles, the contact angle values (approximately 97°) indicated a significant improvement in surface hydrophobicity compared to samples coated with native xylan dispersions containing metal oxide nanoparticles, where the contact angle values ranged between 77° and 83°.

The KIT test results indicated that both xylan acetylation and nanoparticle incorporation improved the grease resistance of the coated paper. Samples coated with acetylated xylan and ZnO nanoparticles exhibited a stronger positive effect compared to those containing CuO nanoparticles.

The combination of acetylated xylan with ZnO and CuO nanoparticles resulted in a moderate antibacterial activity against *Bacillus* sp. and more persistent inhibitory effect on sporulation of *Penicillium* conidia, suggesting a synergistic interaction between surface hydrophobicity and nanoparticle-induced oxidative stress.

This study demonstrates that imidazolium-based ionic liquids, particularly [Emim]Ac, represent an efficient and environmentally friendly alternative for the acetylation of xylan hemicelluloses. The developed coatings showed promising properties for sustainable food-packaging applications. However, additional results regarding migration behavior, food-contact performance, and large-scale implementation, which are part of an ongoing research program, will be reported in future studies.

## 5. Patents

Patent request no. WO2025216645-A1, Composite mixture based on hemicellulose esters, process for obtaining it, and testing the specific properties of packaging for agri-food products.

## Figures and Tables

**Table 1:** Physico-mechanical properties of base paper.

Characteristic, [M.U.]	Value
Grammage, [g/m^2^]	50.48 ± 0.15
Thickness, [mm]	0.710 ± 0.007
Gurley porosity, [s/100 mL]	90 ± 4.04
Bursting strength, [KPa]	177 ± 8.74
Water absorption (Cobb Index), [g/m^2^]	27 ± 1.15
Contact angle, [°]	63.78 ± 1.04
Oil and grease resistance, [KIT test]	3 ± 0.58

**Table 2:** Samples codification and the composition of coating formula.

Sample Code	Coating Formulation (%, *w*/*w*)			
	Native Xylan	Acetylated Xylan with [Emim]Ac	ZnONPs (*)	CuONPs (*)
(Xy)	100	-	-	-
(XyZnO10)	100	-	10	-
(XyZnO20)	100	-	20	-
(XyCuO10)	100	-	-	10
(XyCuO20)	100	-	-	20
(AcXy)	-	100	-	-
(AcXyZnO10)	100	10	-	-
(AcXyZnO20)	100	20	-	-
(AcXyCuO10)	100	-	10	-
(AcXyCuO20)	100	-	20	-

(*) The nanoparticle content was expressed as weight percentage relative to the dry mass of xylan used in the coating formulation.

**Table 3:** Antibacterial activity of paper samples coated with composite mixtures based on xylan hemicellulose and metal oxide nanoparticles against the *Bacillus subtilis*.

Sample Descriptions	Degree of Colony Growth (*), After		Inhibition Degree, [%], After	
	24 h	48 h	24 h	48 h
Xy (paper coated with native xylan)	(+ + +)	(+ + +)	00.00	0.00
			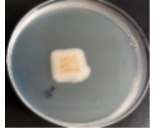	
XyZnO10 (paper coated with native xylan+ 10%ZnONPs)	(+ + −)	(+ + −)	60 ± 0.19	30 ± 0.17
			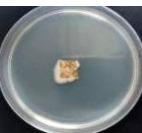	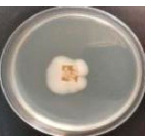
XyZnO20 (paper coated with native xylan+ 20%ZnONPs)	(− − −)	(− − −)	100 ± 0.00	100 ± 0.00
			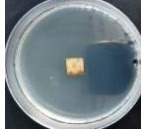	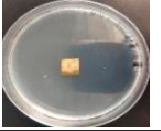
XyCuO10 (paper coated with native xylan+ 10%CuONPs)	(+ + −)	(+ + +)	20 ± 0.22	15 ± 0.12
			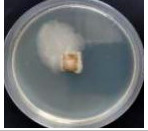	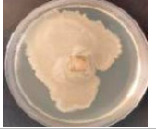
XyCuO20 (paper coated with native xylan+ 20%CuONPs)	(+ − −)	(+ + −)	60 ± 0.00	35 ± 0.00
			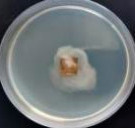	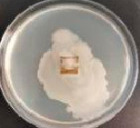
AcXy (paper coated with [Emim]Ac acetylated xylan)	(+ + −)	(+ + −)	40 ± 0.18	40 ± 0.12
			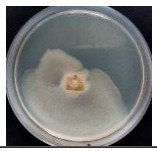	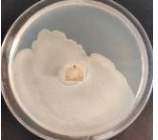
AcXyZnO10 (paper coated with [Emim]Ac acetylated xylan + 10%ZnO)	(+ − −)	(+ + −)	66 ± 0.23	35 ± 0.16
			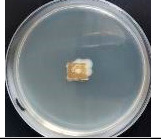	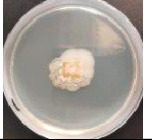
AcXyZnO20 (paper coated with [Emim]Ac acetylated xylan + 20%ZnO)	(+ + −)	(+ + +)	50 ± 0.13	15 ± 0.12
			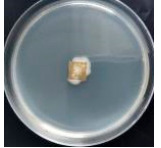	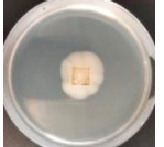
AcXyCuO10 (paper coated with [Emim]Ac acetylated xylan + 10%CuO)	(+ − −)	(+ − −)	66 ± 0.21	66 ± 0.21
			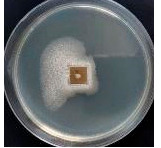	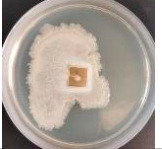
AcXyCuO20 (paper coated with [Emim]Ac acetylated xylan + 20%CuO)	(+ − −)	(+ + −)	66 ± 0.14	45 ± 0.11
			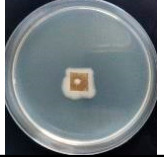	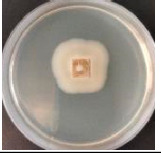

(*) very good growth (+ + +), good growth (+ + −), poor growth (+ − −), and no growth (− − −).

**Table 4:** Papers coated with xylan dispersions and metal oxide nanoparticles: Cultural characteristics and inhibition degree for *Penicillium*.

Sample Codification	Inhibition Degree, %, After			
	3 Days	7 Days	14 Days	21 Days
Xy	0.0	0.00	0.00	0.00
	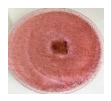	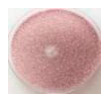	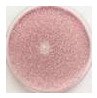	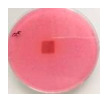
XyZnO10	0.00 *	0.00	0.00	0.00
	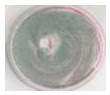	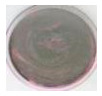	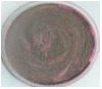	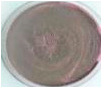
XyZnO20	0.00 *	0.00	0.00	0.00
	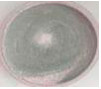	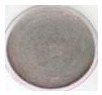	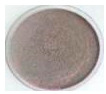	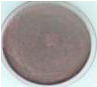
XyCuO10	0.00	0.00	0.00	0.00
	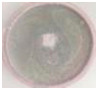	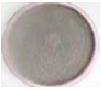	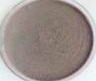	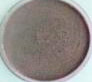
XyCuO20	0.00	0.00	0.00	0.00
	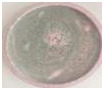	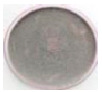	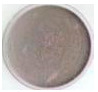	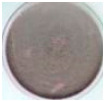
AcXy	0 *	0	0	0
	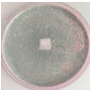	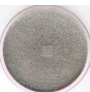	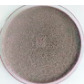	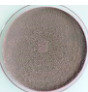
AcXyZnO10	0 *	0 *	0 *	0 *
	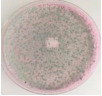	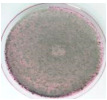	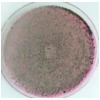	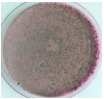
AcXyZnO20	0 *	0	0	0
	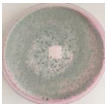	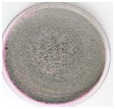	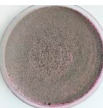	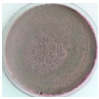
AcXyCuO10	0 *	0 *	0 *	0 *
	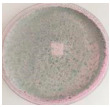	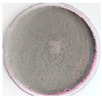	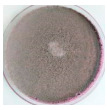	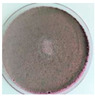
AcXyCuO20	0 *	0	0	0
	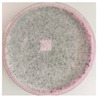	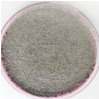	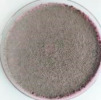	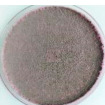

* Inhibitory effect on sporulation.

## Data Availability

Data are contained within the article.

## Abbreviations

The following abbreviations are used in this manuscript:

ZnONPs	Nanoparticles of ZnO
CuONPs	Nanoparticles of CuO
[Emim]Ac	1-ethyl-3-methylimidazolium acetate
FT-IR	Fourier-transform infrared spectroscopy
^1^H-NMR	Proton nuclear magnetic resonance
SEM	Scanning electron microscopy
TGA	Thermogravimetric analysis
DS	Substitution degree
